# An Important Meeting

**Published:** 1893-06

**Authors:** 


### AN IMPORTANT MEETING.

THE National Conference of the State Medical Examining and Licensing Boards will convene in annual session for the third time, at the Pfister Hotel, Milwaukee, on Tuesday, June 7, 1893.

This organization has grown out of the conditions that are obtaining in the several States that require a separate State license, before conferring the right to practise medicine on any individual. Year by year the numbers of these commonwealths are increasing, and at the rate of the increase now going on it will not be long before every State will require a separate examination for a medical license to practise.

There can be no question as to the good influence these laws are exerting in improving professional *esprit de corps*, and in elevating professional standards throughout the country. The existence of these boards stimulates teaching faculties to do better work, and, year by year, the ratio of rejections will naturally decrease.

At the annual conference of the Medical Examining and Licensing Boards, practical questions are considered relating to the methods of holding examinations, a uniformity in which seems very desirable. There should be a reciprocity between the States in this matter, so that a physician holding a license in one State may register in another, on removal thereto, without undergoing an additional examination. This reciprocity can only be brought about by having the standards uniform and of equal merit.

There is no organization in the country capable of exerting a greater influence for good upon the profession of medicine, and directly and indirectly upon the sanitation of the country, than this National Conference of State Examining and Licensing Boards.

While the members of the conference are limited to active and retired members of examining boards, yet all persons interested are invited to the meeting to exchange views with the members, and to assist in perfecting the scheme for improved educational standards in all the States.

A number of papers will be read at this meeting in accordance with the schedule announced in the May number of the JOURNAL, and discussions will be held upon these and other topics that will prove of interest and value to all who attend. It is to be hoped that every State board will be represented at the conference.

